# Utility and Acceptability of a Brief Type 2 Diabetes Visual Animation: Mixed Methods Feasibility Study

**DOI:** 10.2196/35079

**Published:** 2022-08-09

**Authors:** Mohsen Alyami, Anna Serlachius, Mikaela Law, Rinki Murphy, Turky H Almigbal, Mataroria Lyndon, Mohammed A Batais, Rawabi K Algaw, Elizabeth Broadbent

**Affiliations:** 1 Department of Psychological Medicine Faculty of Medical and Health Sciences The University of Auckland Auckland New Zealand; 2 School of Medicine Faculty of Medical and Health Sciences The University of Auckland Auckland New Zealand; 3 Auckland District Health Board Auckland New Zealand; 4 Department of Family and Community Medicine College of Medicine King Saud University Riyadh Saudi Arabia; 5 King Saud University Medical City King Saud University Riyadh Saudi Arabia; 6 Centre for Medical and Health Sciences Education Faculty of Medical and Health Sciences The University of Auckland Auckland New Zealand; 7 Vision College of Medicine Vision Colleges Riyadh Saudi Arabia

**Keywords:** illness perception, visualization, animation, intervention, mobile phone, type 2 diabetes mellitus

## Abstract

**Background:**

Visualizations of illness and treatment processes are promising interventions for changing unhelpful perceptions and improving health outcomes. However, these are yet to be tested in patients with type 2 diabetes mellitus (T2DM).

**Objective:**

This study assesses the cross-cultural acceptability and potential effectiveness of a brief visual animation of T2DM at changing unhelpful illness and treatment perceptions and self-efficacy among patients and family members in 2 countries, New Zealand and Saudi Arabia. Health care professionals’ views on visualization are also explored.

**Methods:**

A total of 52 participants (n=39, 75% patients and family members and n=13, 25% health care professionals) were shown a 7-minute T2DM visual animation. Patients and family members completed a questionnaire on illness and treatment perceptions and self-efficacy before and immediately after the intervention and completed semistructured interviews. Health care professionals completed written open-ended questions. Means and 95% CIs are reported to estimate potential effectiveness. Inductive thematic analysis was conducted on qualitative data.

**Results:**

All participants rated the visual animation as acceptable and engaging. Four main themes were identified: animation-related factors, impact of the animation, animation as an effective format for delivering information, and management-related factors. Effect sizes (ranged from 0.10 to 0.56) suggested potential effectiveness for changing illness and treatment perceptions and self-efficacy among patients and family members.

**Conclusions:**

Visualizations are acceptable and may improve the perceptions of patients’ with diabetes in a short time frame. This brief visual animation has the potential to improve current T2DM education. A subsequent randomized controlled trial to investigate the effects on illness and treatment perceptions, adherence, glycemic control, and unplanned hospital admission is being prepared.

## Introduction

### Background

Type 2 diabetes mellitus (T2DM) is a metabolic condition characterized by high blood sugar levels owing to a loss of pancreatic beta cell function [1]. An estimated 463 million people had diabetes worldwide in 2019, and T2DM accounts for 90%-95% of all diabetes cases [2]. In New Zealand, 5.5% of people aged ≥15 years have diabetes [3]. Māori (indigenous New Zealanders) and Pacific groups are disproportionately affected by diabetes, with the latest report showing the prevalence rate of diabetes is 1.8 and 2.6 times higher in Māori and Pacific groups than non-Māori and non-Pacific groups [3]. In Saudi Arabia, the incidence rate of diabetes has substantially increased over the past 30 years, and the prevalence of diabetes was 18.3% in adults aged 20-79 years in 2019 [4].

T2DM requires ongoing self-management and lifestyle changes to achieve glycemic control and minimize the risk of complications [4]. Patients with T2DM are recommended to engage in a range of self-care behaviors, including taking medications as prescribed, following a healthy diet, and being physically active. Other self-care behaviors include blood glucose testing, foot care, smoking cessation, and attending clinic appointments [5].

However, research has shown that low adherence to diabetes self-care behaviors is common [6], and approximately 50% of patients with T2DM in New Zealand and Saudi Arabia do not reach glycemic control (HbA_1c_) targets [7,8]. Ethnic disparities in T2DM management exist, where the Māori and Pacific groups are at greater risk of exhibiting lower adherence to metformin (first-line glucose-lowering oral medications; [6]), higher HbA_1c_ levels [9], and experiencing more diabetes-related complications compared with New Zealand European groups [8]. This is concerning because low adherence is associated with suboptimal glycemic control, increased medical cost, hospitalization, and mortality [10,11].

There is a growing awareness of the importance of psychosocial factors in the management of diabetes, as highlighted by recommendations to integrate psychosocial support into routine diabetes care [12]. Evidence-based psychological interventions for patients with diabetes include illness-perception interventions [13,14]. These interventions are based on the common sense model (CSM) [15,16], in which patients are active problem-solvers who use coping strategies based on their cognitive and emotional representations of illness. Research has demonstrated that these mental representations (known as illness perceptions) are associated with health outcomes [17-22]. The central dimensions include perceptions of the identity, timeline, causes, consequences, and control of illness.

Related to CSM is the necessity-concerns framework, which proposes that patients also have beliefs about medicine and that these beliefs influence adherence to treatment [23,24]. Patients who perceive medicine as necessary and have fewer concerns about its side effects are more likely to adhere to medication [24].

In diabetes, systematic reviews have established that patients’ perceptions of personal control over their illness are associated with better glycemic control, whereas greater illness identity perceptions (attributing more symptoms to diabetes), greater consequences perceptions (perceiving diabetes to have severe consequences), higher emotional distress, and concern perceptions about diabetes were associated with suboptimal glycemic control [17,22]. Illness perceptions have also been linked to other outcomes in diabetes, including adherence to self-care behaviors [25], quality of life, and depressive and anxiety symptoms [26]. Similarly, evidence from reviews shows that higher necessity beliefs and fewer concerns about side effects were associated with higher adherence to medicines across studies [27,28].

Illness perceptions of family members can also influence patients’ health behaviors, as many self-care behaviors in diabetes occur within patients’ social contexts. Patients with chronic conditions tend to have better health outcomes when their perceptions align with those of their family members [29]. In diabetes research, mediation studies have shown that partners’ perceptions can mediate the effects of patients’ perceptions on adherence to self-care behaviors [30,31]. For example, a study with recently diagnosed patients with T2DM and their partners found that partners’ personal control and treatment control perceptions fully mediated the effects of patients’ perceptions on their adherence to blood glucose testing (mediation effects of 0.050 and 0.095, respectively). Partners’ consequence perceptions also mediated the effects of patients’ consequence perceptions on their adherence to exercise, foot care, and blood glucose testing, with mediation effects ranging from −0.052 to 0.062 [30]. This research highlights the importance of family members’ perceptions of diabetes and the need to involve family members or significant others when designing interventions to improve patient health outcomes [32].

Research suggests that addressing unhelpful illnesses and treatment perceptions can result in better coping behaviors and improved health outcomes [29]. Interventions to change illness perceptions have shown promising results in patients with T2DM. For example, a family-based intervention resulted in improved understanding of T2DM, increased perceptions of personal and treatment control, fewer symptoms attributed to T2DM, and decreased concerns and emotional distress [14]. This intervention also improved adherence to diet, exercise, self-efficacy, well-being, and family support at 6-month follow-up [14]. Other similar interventions have resulted in improved adherence to self-care behaviors [13]. Although these interventions improve diabetes management and other psychological outcomes, they are time-consuming and are often conducted over multiple sessions. Owing to short medical consultations and constrained health budgets, illness perception interventions need to be more scalable to increase clinical utility [33]. The inclusion of visualization (eg, an animated pictorial explanation) of illness and its treatment could reduce delivery time.

Robust theoretical models (eg, cognitive theory of multimedia learning) and published empirical studies support the use of visuals to improve learning [34,35]. In health psychology research, visualizations have been shown to be an effective medium to deliver information about illnesses and treatment processes, help explain abstract concepts, and show how treatment processes work inside the body [36]. Other benefits are that visualizations can be brief (eg, 10-15 minutes), improve visual appeal, do not require advanced training to deliver, and can be delivered in all settings (eg, in person or on the web; [33,37-39]). Research has shown that visualizations improve not only perceptions but also health behaviors [37,40]. For example, visual interventions with acute coronary syndrome, osteoporosis, HIV, and oncology patients have been shown to improve understanding and control perceptions in a short timeframe [33,38], increase adherence to antiretroviral therapy [41,42], increase exercise and improve return to normal activities [33], and improve postoperative mobility [43]. This research suggests that visualization can improve patients’ perceptions and health-related behaviors in a number of conditions.

### Objectives

Research on the effects of visualization on illness and treatment perceptions of T2DM is lacking. Therefore, this pilot study aimed to explore the cross-cultural acceptability of a brief visual animation of T2DM among patients and family members across 2 countries and collect feedback from health care professionals (HCPs) to highlight ways in which the visual animation could be improved. The study also assessed potential effects of the visual animation on illness and treatment perceptions and self-efficacy to inform a future trial on adherence to medication, diet and exercise behaviors, and health outcomes (eg, glycemic control and unplanned hospital admissions).

## Methods

The authors followed the Standards for Reporting Qualitative Research [44] and CONSORT (Consolidated Standards of Reporting Trials) guidelines for pilot studies [45].

### Design and Sample Size

This pilot study used a mixed methods design, involving pre-post assessment and semistructured interviews with patients with T2DM and family members across 2 countries (New Zealand and Saudi Arabia). This study also explored views about the visual animation among HCPs in New Zealand using open-ended questions. Given its exploratory nature, this study was not powered to detect statistical significance; however, it assessed potential effects on illness and treatment perceptions and self-efficacy by looking at changes in mean scores from before to after the intervention. The effect sizes were calculated from the means and SDs, which may be useful for a power calculation for a future trial. A previous study using visual animation found small effect sizes for illness identity perceptions and return to normal activities in patients with acute coronary syndrome [33], but effect sizes may differ in this population. To meet the study aims, we planned to recruit 32 patients (16 patients from each county) and as many family members as possible.

### Participants

Primary participants were patients with T2DM. Patients were eligible to participate if they were aged ≥18 years, had a formal diagnosis of T2DM for ≥1 year, were prescribed diabetes medications, lived in New Zealand (for the New Zealand participant group) or Saudi Arabia (for the Saudi Arabia participant group), and had access to the internet and a smartphone or computer. Eligible participants were encouraged to invite their family members to participate in the study. A family member was defined as a relative in regular contact with a person with T2DM. Participating family members had to be aged ≥18 years, living in New Zealand or Saudi Arabia, and with access to the internet and a smartphone or computer. Patients were allowed to participate by themselves if they did not want to invite a family member. Family members were also allowed to participate by themselves if they found it more convenient for them.

HCPs were consulted for feedback on the visual animation. In addition to working at an outpatient diabetes clinic, there were no other inclusion or exclusion criteria for this group. All participants were recruited between March and July 2021.

### Brief Visual Animation of T2DM

The brief visual animation was developed by a multidisciplinary team including health psychologists, endocrinologists, and developers. The developmental process involved iterative feedback from the multidisciplinary team to refine the visual content. Māori and Pacific HCPs were consulted to ensure cultural appropriateness.

The visual animation is a 7-minute video that begins with introductory statements explaining the focus and purpose of the visual animation. The visual animation shows the production of glucose in the body after food consumption, glucose levels in the blood, and how glucose and insulin interact to allow glucose to enter body cells using the lock-and-key analogy. The visual animation then depicts what happens when patients have T2DM (eg, glucose cannot enter body cells because of inadequate insulin or insulin resistance, which leads to increased glucose levels in the blood). Symptoms and long-term complications associated with T2DM are visually depicted. The visual animation shows how treatment (with a particular focus on metformin, healthy eating, and regular exercise) can help control blood glucose levels. The visual animation concludes with an emphasis on the importance of family and significant others as a source of support and motivation. We developed 2 versions of the visual animation, one in English suited for the New Zealand context and one in Arabic suited for the Saudi context. Differences included the appearance and dress of the characters, food depicted, and pictures of the environment when the character was exercising outside (Figures S1-S11, Multimedia Appendix 1).

### Procedure

Participants were recruited from an outpatient diabetes clinic at the Greenlane Clinical Centre in Auckland, New Zealand, a specialized diabetes clinic at a tertiary hospital in Riyadh, and Facebook diabetes support groups and community groups. Patients and their family members were approached in the waiting rooms by a student researcher (New Zealand sample) or a medical intern (Saudi sample) who introduced the study and invited them to participate. A study flyer was posted on community and diabetes support pages on Facebook outlining brief information about the study and the research team contact details. Interested participants were provided with a link to the study on Qualtrics (web-based software for data collection; [46]).

On Qualtrics, participants confirmed their eligibility, viewed and downloaded participant information sheets, provided web-based consent, completed baseline questionnaires, and chose a time for the interview. At the beginning of each semistructured interview, participants were shown the brief visual animation either on web using Zoom software (New Zealand sample) or face-to-face at a clinic in an office room (Saudi sample). The participants then completed a web-based questionnaire immediately after the intervention. Participants were then interviewed, and only the participants and interviewers were present. All interviews were audio recorded and lasted for up to 60 minutes (Multimedia Appendix 2 provides the interview schedule). As a token of appreciation for their time, each participant in the New Zealand sample received a NZ $50 (US $31) voucher. The participants in the Saudi patient sample did not receive compensation.

The interviews were conducted by the first author (MA), a male PhD health psychology student originally from Saudi Arabia but studying or residing in New Zealand, and a female medical intern living in Riyadh, Saudi Arabia. The interviewers had no relationships with the participants before the commencement of the study.

HCPs working at an outpatient diabetes clinic at the Greenlane Clinical Centre in Auckland, New Zealand, were asked to provide feedback about the visual animation. The first author (MA) gave a presentation during the clinic staff meeting and showed the brief visual animation of T2DM. HCPs responded to 7 written open-ended questions related to the visual animation (Multimedia Appendix 2 provides the open-ended questions).

### Measures

Patients with T2DM provided their age, sex, ethnicity, marital status, educational level, partnership status, employment status, type of prescribed diabetes medications, and duration of T2DM. Family members also provided information on age, sex, ethnicity, educational level, and relationship with the patient.

At baseline, patients’ perceptions of T2DM were assessed using 4 items of the Brief Illness Perception Questionnaire (B-IPQ; [47]). The items included consequence, personal control, coherence, and concern perceptions. These 4 items were chosen as they have been consistently identified to be related to outcomes in diabetes (eg, glycemic control; [17,22], and perceptions of personal control and coherence are among the most frequently changed perceptions in trials using CSM in diabetes [32]. These perceptions were targeted in the intervention. These items were scored on a scale from 0 to 10, with higher scores indicating stronger perceptions. The B-IPQ has been previously used with Māori, Pacific, and Saudi patients with T2DM [48,49] and with family members [50]. The B-IPQ has demonstrated robust psychometric properties [17,47].

Patients’ perceptions regarding the effectiveness of treatment (medication, healthy eating, and regular exercise) in controlling T2DM were assessed using 3 items adapted from previous research ([25]; eg, *How much do you think your medication can help control your diabetes?*). Patients’ confidence in managing their T2DM (self-efficacy) was evaluated using a single item (*How confident do you feel in managing your diabetes?*). These items were scored on a scale from 0 (not helpful or not confident at all) to 10 (extremely helpful or extremely confident). Patients’ beliefs about the necessity of taking diabetes medications daily were assessed using a single yes or no question. Participants were asked to explain their answer as to whether they perceived it necessary to take diabetes medication every day.

Immediately after watching the visual animation, patients completed the same questionnaire administered at baseline. Participants further completed 3 yes or no questions related to their perceptions of T2DM (eg*, Did the animation make you think about [the potential consequences of your diabetes, [things you could do to help control your diabetes, and [your diabetes medication]*?). These questions were designed to assess whether the visual animation prompted the patients to actively think about their T2DM.

Family members completed similar questionnaires at baseline and immediately after viewing the visual animation; however, the questions were slightly modified to ask about their perceptions of their family members’ T2DM (eg, *How much control do you feel your family member has over their diabetes?*).

### Data Analysis

#### Qualitative Data

Interviews with Saudi patients were translated and transcribed into English by an independent researcher from Saudi Arabia who held a bachelor’s degree in English literature. The transcriptions were then checked against the original recordings for accuracy by the first author (MA), who was fluent in Arabic and English. Interviews with patients from New Zealand and family members were transcribed by the first author (MA). Transcriptions were emailed to participants who wished to review their interview transcriptions and were instructed to return any comments within 2 weeks. All qualitative data from interviews and responses to open-ended questions provided by HCPs were coded and analyzed using an inductive thematic analysis approach [51]. Thematic analysis and coding were conducted independently by 2 researchers: the first author (MA) and a female health psychology researcher (ML) experienced in thematic analysis. Both researchers followed the 6 phases of thematic analysis [51].

First, data familiarization was achieved through manually transcribing, reading, and rereading the data. During this phase, a list of initial ideas regarding the data set was generated. Second, the entire data set was coded and each code was matched to the data extracts. Third, the generated codes were sorted and combined to form the initial themes and subthemes, and all relevant coded data extracts were collated within each initial theme and subtheme. All coded data extracts were reviewed to ensure coherence and meaningfulness. Themes were then further refined in relation to the entire data set to ensure that each theme was distinct. Themes and subthemes were assigned labels that captured their essence. Discussions between the researchers ensued until a consensus was reached on the themes and the strongest quotes to support each theme and subtheme.

#### Quantitative Data

Descriptive statistics were used to analyze the data. Frequencies and percentages were calculated for dichotomous data. Continuous data were summarized using the mean and 95% CI at baseline and immediately after the intervention. Effect size (*r*) was calculated and interpreted using Cohen’s guidelines (*r*=0.1, small; *r*=0.3, medium; and *r*=0.5, large) [52] to determine the sample size calculation for a subsequent trial. All the analyses were performed using SPSS (version 27; IBM Corp).

### Ethics Approval

This study was reviewed and approved by the Auckland Health Research Ethics Committee (reference number AH3217) and the General Directorate of Health Affairs, Najran Institutional Review Board, Saudi Arabia (IRB 20‐040E).

## Results

### Overview

**Figure 1:**
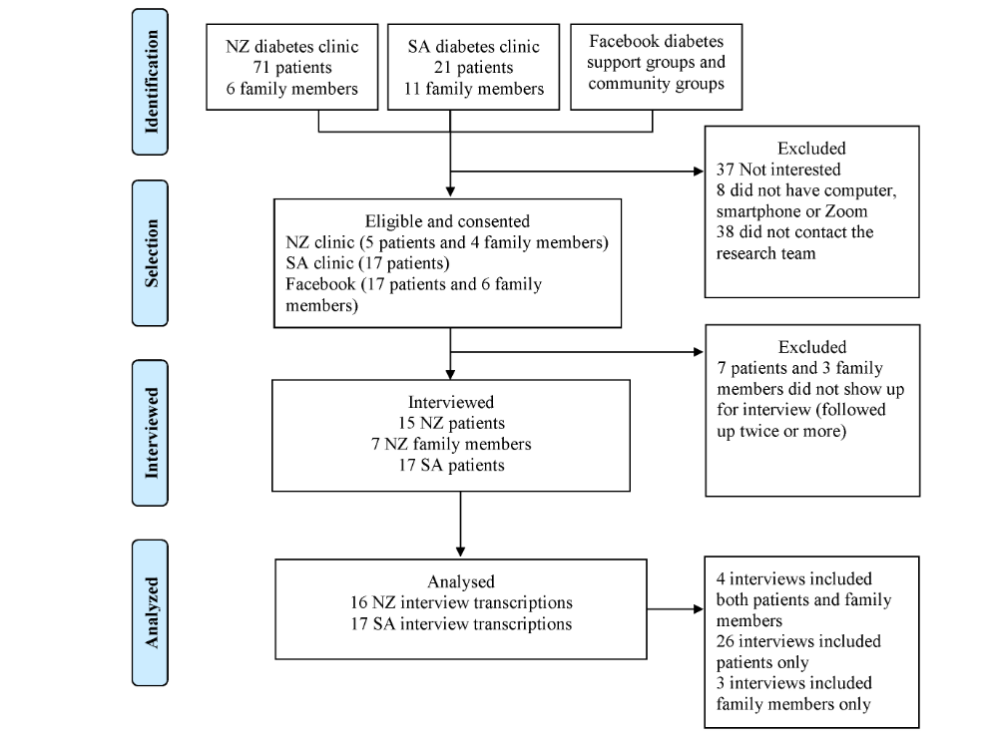
Participant flowchart. NZ: New Zealand; SA: Saudi Arabia.

There were 52 participants (n=15, 29% New Zealand patients; n=17 (33%) Saudi patients; n=7, 13% New Zealand family members; and n=13, 25% HCPs). Figure 1 provides the participant flowchart. Mean age of the participants was 54 (SD 11.16) years for patients and 44.1 (SD 11) years for family members. Patients had been living with T2DM for an average of 9 (SD 7.09) years. Table 1 shows the participant characteristics. The participating family members included 1 parent, 3 spouses, 2 children, and 1 other family member of the patient with T2DM. Most HCPs were diabetes nurses (6/13, 46%), followed by medical and nursing students (4/13, 31%), doctors (2/13, 15%), and a diabetes care coordinator (1/13, 8%).

**Table 1:** Demographic and clinical characteristics of the sample.

Characteristics		NZ^a^ patients (n=15)	SA^b^ patients (n=17)	NZ family members (n=7)
Age (years), mean (SD)		55.5 (11.1)	52.7 (11.4)	44.1 (11.0)
Female, n (%)		10 (67)	9 (53)	6 (86)
**Ethnicity, n (%)**				
	NZ European	7 (47)	0 (0)	3 (43)
	Māori	3 (20)	0 (0)	0 (0)
	Cook Island	1 (7)	0 (0)	0 (0)
	Niuean	0 (0)	0 (0)	1 (14)
	Indian	1 (7)	0 (0)	2 (29)
	Saudi	0 (0)	15 (88)	0 (0)
	Other	2 (13; Palestinians) and 1 (7; Filipino)	1 (6; Egyptian) and 1 (6; Yemeni)	1 (14; South Asian)
**Education level, n (%)**				
	No formal education	2 (13)	2 (12)	0 (0)
	Secondary education	3 (20)	2 (12)	0 (0)
	High school	4 (27)	4 (23)	1 (14)
	University education	6 (40)	9 (53)	6 (86)
**Partnership status, n (%)**				
	Single	4 (27)	0 (0)	—^c^
	Married or civil union	9 (60)	15 (88)	—
	Couple or de facto	1 (7)	0 (0)	—
	Widowed	1 (7)	2 (12)	—
Working (yes), n (%)		8 (53)	6 (35)	—
Taking metformin (yes), n (%)		13 (87)	14 (82)	—
**Diabetes medications, n (%)**				
	Oral medications only	9 (60)	11 (65)	—
	Insulin therapy only	1 (7)	2 (12)	—
	Oral and insulin	5 (33)	4 (23)	—

^a^NZ: New Zealand.

^b^SA: Saudi Arabia.

^c^Not available.

### Qualitative Findings (Patients and Family Members)

#### Overview

Four main themes were identified from the patients’ and family members’ combined data set: (1) animation-related factors, (2) impact of the animation, (3) animation as an effective format, and (4) diabetes management–related factors. Subthemes have been presented in the text using *italics*. A small number of quotes have been included in the main text to illustrate the major points, and further quotes are provided in Multimedia Appendix 3.

#### Theme 1: Animation-Related Factors

This theme covered participants’ perceptions and views of the visual animation. Participants viewed the visual animation as *succinct and simple*, which provided basic “need to know” information on T2DM. The simplicity and short length of the visual animation helped to keep the participants engaged. Participants highly appreciated this and reported that this amination might be more suitable for people who do not want too much detail or people who are newly diagnosed with T2DM.

Participants reported that a longer visual animation would have probably made them lose interest. A few participants from New Zealand noted that the visual animation was slow with some unnecessary pauses, whereas some family members and participants from Saudi Arabia reported that it was too fast for them.

The use of simple nonmedical language and creative visuals were identified as important elements that made the visual animation *easy to understand*, especially for older adults and those with low health literacy. Participants reported that the visual animation was *informative,* and increased their understanding of T2DM and its management. The participants appreciated that the visual animation not only focused on explaining what T2DM is, its symptoms, and related complications but also on what can be done to help control T2DM. This made them feel that their T2DM was manageable by taking medication as prescribed, eating healthy food, and exercising regularly in addition to other self-care behaviors such as self-monitoring of blood glucose levels and foot care:

I felt I can be more in control now by doing the simple three stuff, take my medication every day, eat healthy and watch my portions and exercise daily.

Saudi female patient 2, 49 years

I suppose we know it’s manageable, what it made me think was I’ve got to make it more manageable.

New Zealand male patient 15, 56 years

Participants identified with the *characters* in the visual animation and said that they were “relatable.” The main character showed emotions (eg, happy and sad), which made them look more realistic than the static pictures found in brochures and pamphlets. More participants from Saudi Arabia identified with the characters compared with participants from New Zealand, and this might be because New Zealand is more multicultural than Saudi Arabia; therefore, it is harder to make the animated characters relatable for all cultures in New Zealand.

Participants highlighted the importance of making the characters context-specific, where characters reflect culture. This made the visual animation feel inclusive of minority groups such as the Māori and Pacific groups in New Zealand who are at higher risk of developing T2DM. Participants from Saudi Arabia also appreciated that the visual animation was custom-made to suit their cultural context.

Some participants felt that they would relate more to the animated characters if they were personalized to match participants’ demographics. However, others acknowledged that diabetes can affect anyone regardless of ethnicity or gender and said that personalizing the animated characters was unnecessary.

#### Theme 2: Impact of the Animation

This theme was related to the perceived impact of the visual animation, in which *participants reflected on their’ and their family members’* T2DM. The visual animation made patients think about the status of their T2DM (eg, whether controlled or uncontrolled) and the importance of adhering to the treatment regimen. For some participants, the visual animation was “a wake-up call” that reinforced the fact that diabetes is a chronic illness and the potential consequences of uncontrolled diabetes and motivated them to make positive lifestyle changes:

It is sort of a bit of a wakeup call now that I can understand it better. Sort of how important it is to follow that [lifestyle changes].

New Zealand female patient 5, 62 years

For others, the visual animation reinforced what they had been told in the past about the importance of self-care behaviors and reassured them that diabetes medications work to control their blood glucose levels. Other participants who managed their T2DM well felt reassured that they were on the right track. A few participants reflected on their low adherence to diabetes self-care behaviors and reported feelings of guilt.

Of note, participants from Saudi Arabia reported that the visual animation specifically made them think about the potential complications of T2DM and the need to improve their adherence to delay or avoid these events, whereas participants from New Zealand did not. Participants from New Zealand reported general concerns about T2DM-related complications, but this was not related to the visual animation itself:

I thought about the consequences of diabetes, especially on the body organs. I felt that if I really do not control my diabetes, I will suffer more, and I will have more serious complications than I have so far.

Saudi female patient 6, 59 years

For family members, the visual animation made them reflect on what they were already doing and what they could do to better support the patient, including preparing healthier meals and reminding them to take their medications. For those who lived apart from the patient, there was some uncertainty about whether the patients were, in fact, adherent to self-care behaviors:

That worked well for him, cutting down his portions yeah. It gives you a sense of control. That’s what you should be eating and aiming for.

New Zealand female family member 19, 58 years

Family members reported a sense of pride about their relatives with T2DM who were making significant lifestyle changes to control their T2DM. They were seen as role models who could influence other family members to have a better lifestyle, including improving physical activity and watching the type and portions of their food:

I was thinking about how he’s doing all that like every day, whether it rains or shines, he gets up and goes for his exercise. And even the food, even if he likes certain food, he restricts the amount that he eats, which also makes other people in the family realize OK you have to control your portion of food that you’re eating even if you really love it.

New Zealand female family member 16, 57 years

#### Theme 3: Animation as an Effective Format

This theme covered the participants’ perceived reasons for why the visual animation was an effective format. Information that participants had come by or received in the past (either written or verbal) was often described as confusing and medical. Many participants reported not reading diabetes pamphlets available at clinics and pharmacies, and those who read them thought they were often limited and primarily diet based. The visual animation was regarded as superior to written information as *visuals helped participants conceptualize T2DM and its treatment processes*. Some abstract thinking is needed to understand T2DM, and the participants acknowledged the value of being able to “see inside the body, ” what it meant to have T2DM, the role of insulin, and how metformin works inside the body. Visual information is easier to understand and retrieve from the memory. Participants perceived the visual animation as more engaging compared with other forms, such as written information. The visual animation was also regarded as culturally responsive to people with limited education, low health literacy, and diabetes-related vision problems:

We have seen pamphlets, but we often throw them out. They are hard to understand and too many words...I think the video is much better because you can see inside the body. I knew things before, but in my mind, I never pictured it. Now, I know how insulin works.

New Zealand male patient 8, 71 years

It is powerful, you know, seeing how food is broken down into sugar, going through the bloodstream, and used for energy. How insulin is essential for this process and without it, like in type 1 diabetes, you can die.

Saudi male patient 11, 43 years

It is culturally responsive to a whole section of society that might find it valuable, because it’s not just written literature, that it is very medical with medical speak. I think that the visual aspect is very good.

New Zealand female family member 3, 48 years

Despite having been living with T2DM for years, the animation presented *new information* for some participants. For example, using the lock-and-key analogy to explain the role of insulin was new to most participants. Participants reported having not previously been told about some of the explanations covered in the brief visual animation, including how metformin and insulin work inside the body. In some instances, there was a complete lack of knowledge about self-testing of blood glucose and foot care despite the importance of these behaviors. Participants reported that the visual animation helped bridge this knowledge gap and motivated them to discuss these issues further with their care team:

It is more than what I have been told by anybody else basically...I had no clue about that, testing your sugar levels daily!

New Zealand female patient 2, 61 years

I got to know more about diabetes, most of which I hadn’t known previous to watching the video.

Saudi female patient 13, 60 years

#### Theme 4: Diabetes Management–Related Factors

This theme covered general issues around diabetes management (knowing and doing are 2 different things), which were not related to the visual animation itself. Participants identified main *barriers to adherence* to medication, healthy eating, and exercise, including poor understanding of the chronicity and progression nature of diabetes, forgetfulness, older age, intentionally skipping doses as they felt okay, busy work or personal schedules, physical disability, poor weather, lack of willpower and motivation, and stress.

Participants expressed *concerns* and *frustrations* related to diabetes management. These included concerns about medication side effects, especially insulin, such as weight gain. A few participants reported hesitancy about going on insulin despite being asked a few times by their doctors. This was in part because of poor understanding of the progressive nature of diabetes, and going onto insulin was seen as a sign of personal failure to control diabetes:

I’ve actually had an aversion to the idea of insulin, but before this meeting, I was saying to myself if I go on insulin, then it’s over for me basically.

New Zealand male patient 10, 36 years

Participants expressed frustration about the cost and funding for new diabetes medications such as empagliflozin (Jardiance). This medication is funded in New Zealand only for people with T2DM if they fulfill various eligibility criteria such as being at high risk of heart and kidney complications. Patients who wanted to go onto this new medication but did not meet the eligibility criteria chose to pay the cost themselves, creating financial strain and additional concerns. Another frustration patients and family members experienced was the dismissal by HCPs in instances where patients came to their appointments having done some research on the internet and prepared their questions.

Other participants were concerned about long-term complications if they did not control their T2DM. For some participants, understanding the chronicity and progressive nature of T2DM, in addition to being fearful of potential complications, pushed them to improve their adherence and make serious lifestyle changes.

*The impact of culture*, especially around eating habits, was highlighted as a factor that could hinder people’s ability to manage their T2DM. In some instances, it is considered rude and unacceptable to refuse food, especially when invited to someone else’s house.

*Support from family members and significant others* was seen as important for optimal management. Family members can remind patients to take medications and motivate them to eat healthily and exercise regularly. Participants acknowledged the social impact of T2DM and that caring for someone with T2DM could be highly stressful. One way to mitigate this is through a better understanding of T2DM.

Problems can arise when patients do not want help or live alone, or when family members are not supportive for any reason, including low health literacy. Participants expressed frustration when, for example, family members cooked unhealthy meals or perceived T2DM as something that is easily fixed and not chronic.

### Qualitative Findings (HCPs)

Inductive thematic analysis of the HCPs’ data set resulted in a single theme consistent with the first theme identified from the patients’ and family members’ data set, “animation-related factors.” HCPs perceived the visual animation as *brief and succinct*, e*asy to understand, informative, and culturally appropriate* (Multimedia Appendix 4 provides themes, subthemes, and supporting quotes).

### Suggested Changes to the Visual Animation

Participants from all groups provided suggestions for improvement, including using a less formal and female voiceover, changing medical terms to lay terms (eg, chronic to long-term, glucose to sugar), personalizing the animated characters based on the patient’s demographics, and adding subtitles to accommodate those with hearing difficulties. The participants also suggested adding greetings in Te Reo Māori and other Pacific languages.

Furthermore, the participants expressed a need for more visual content that covered other important issues in diabetes such as how insulin therapy works, daily self-monitoring of blood glucose, HbA_1c_ testing, and foot care. Brief, simple, and accessible educational digital content in the form of animated videos could be developed for each topic.

### Potential Effects on Illness and Treatment Perceptions and Self-efficacy

Data support the potential effectiveness of the brief visual animation on patients’ perceptions and self-efficacy (Table 2). Increases in means from baseline to immediately after intervention for perceptions of consequences, personal control, coherence, medication effectiveness, and self-efficacy among patients from New Zealand suggest medium to large effects (*r*=0.43-0.56). Changes in mean concern, healthy eating, and perceptions of regular exercise effectiveness were smaller. A similar pattern of positive changes was observed among patients from Saudi Arabia, with medium effects (*r*=0.34-0.47) for increases in personal control perceptions, coherence perceptions, medication effectiveness perceptions, healthy eating effectiveness perceptions, and self-efficacy. Changes in consequences, concerns, and perceptions of regular exercise effectiveness were smaller. Finally, perceptions of family members from New Zealand changed in the expected direction, with effect sizes ranging from small to large (*r*=0.10-0.55).

All patients and family members (100%) believed that it was necessary to take diabetes medication every day at baseline and after the intervention. The cited reasons included controlling blood glucose levels, avoiding and delaying complications, and having a better quality of life. After the intervention, most patients and family members reported that the brief visual animation made them actively think about the potential consequences of T2DM, things they could do to control their T2DM, and diabetes medications.

**Table 2:** Illness and treatment perceptions and self-efficacy scores at baseline and immediately after the intervention.

Variable				Baseline, mean (95% CI)		Immediately after intervention, mean (95% CI)		Effect size (*r*)
**NZ^a^ patients (n=15)**								
	**Illness perceptions**							
		Personal control	5.40 (4.11-6.69)		8.07 (7.03-9.10)		0.52	
		Coherence	6.67 (5.35-7.98)		9.07 (8.42-9.71)		0.56	
		Consequences	7.53 (6.47-8.60)		9.27 (8.69-9.84)		0.43	
		Concerns	7.87 (6.61-9.12)		8.67 (7.98-9.35)		0.27	
	**Treatment perceptions**							
		Medication	7.67 (6.55-8.79)		9.33 (8.88-9.79)		0.48	
		Eating healthy food	8.53 (7.58-9.49)		9.07 (8.42-9.71)		0.19	
		Regular exercise	7.87 (6.46-9.27)		8.93 (8.17-9.70)		0.27	
		Self-efficacy	6.13 (4.73-7.54)		8.40 (7.78-9.02)		0.52	
**SA^b^ patients (n=17)**								
	**Illness perceptions**							
		Personal control	7.18 (5.53-8.82)		8.59 (7.77-9.40)		0.36	
		Coherence	7.65 (6.05-9.24)		9.00 (8.19-9.81)		0.41	
		Consequences	8.00 (6.33-9.67)		9.18 (8.57-9.79)		0.23	
		Concerns	6.88 (5.15-8.62)		5.59 (4.06-7.12)		0.24	
	**Treatment perceptions**							
		Medication	8.12 (6.89-9.35)		9.29 (8.67-9.92)		0.47	
		Eating healthy food	8.18 (6.89-9.46)		9.18 (8.69-9.67)		0.38	
		Regular exercise	8.71 (7.52-9.89)		9.41 (9.09-9.73)		0.24	
		Self-efficacy	7.71 (6.22-9.19)		9.06 (8.47-9.65)		0.34	
**NZ** **family members (n=7)**								
	**Illness perceptions**							
		Personal control	6.71 (3.57-9.86)		8.43 (6.93-9.93)		0.44	
		Coherence	7.14 (5.90-8.39)		8.57 (6.98-10.16)		0.37	
		Consequences	8.86 (7.40-10.31)		9.00 (7.81-10.19)		0.10	
		Concerns	8.00 (5.38-10.62)		6.71 (4.23-9.20)		0.39	
	**Treatment perceptions**							
		Medication	6.71 (4.11-9.31)		9.43 (8.03-10.83)		0.51	
		Eating healthy food	7.86 (5.44-10.27)		9.29 (8.59-9.98)		0.40	
		Regular exercise	8.57 (7.39-9.75)		9.57 (8.84-10.30)		0.55	
		Self-efficacy	6.14 (3.56-8.73)		7.57 (5.66-9.49)		0.23	

^a^NZ: New Zealand.

^b^SA: Saudi Arabia.

## Discussion

### Principal Findings

This pilot study showed that a brief animation was acceptable and engaging for patients with T2DM and their families. Inductive thematic analysis revealed 4 main themes related to the brief animation, the impact of animation, animation as an effective format for delivering information, and diabetes management–related factors. Preliminary analysis showed potential cross-cultural effectiveness for improving illness and treatment perceptions and self-efficacy in all patients and family members, with larger effect sizes observed in the New Zealand patient group than in the Saudi patient group. Room for change in the New Zealand patient group may have been larger given the lower baseline means for many of the perception dimensions. Given the explanatory nature of this study, baseline characteristics were not used in the analysis but could be used in a subsequent trial.

The visual animation was well received. Nearly all patients and their family members reported that the visual animation was informative and understandable. This is consistent with previous studies that have shown that visual interventions (eg, animations covering general information about diabetes, symptoms, risk factors, and management) improved diabetes health literacy [53] and knowledge about foot care [54].

Visualizing both the illness and its treatment are important [36]; this is because visualizing T2DM, related symptoms, and serious complications can potentially increase anxiety and fear. Heightened negative emotions alone are not sufficient to elicit changes in perceptions and behaviors; therefore, information on how T2DM can be managed is essential. This visual animation explained T2DM and how treatment (eg, metformin) works inside the body to help control T2DM, which was highly appreciated by the participants. Participants felt that their T2DM was more manageable and increased their control perceptions, self-efficacy, and motivation to make lifestyle changes. This finding is in line with previous research that showed visualizations can increase the motivation to for taking medicine in patients with osteoporosis [38].

Many of the reported barriers to adherence to self-care behaviors (eg, poor understanding, forgetfulness, and concerns about medication side effects and costs) are similar to previous findings [55]. One additional barrier is known as “psychological insulin resistance,” where patients are hesitant to initiate and or adhere to insulin therapy [56-59]. This barrier represents a complex set of beliefs (eg, sense of failure and incompetency) and attitudes (eg, fear of injections and hypoglycemia) toward insulin therapy [56].

Some participants reported avoiding going onto insulin therapy despite the necessity as going onto insulin was seen as a sign of personal failure for not being able to control their T2DM through oral medications and other self-care behaviors. This phenomenon is not uncommon in the literature [60]. This was reported by patients from New Zealand but not by patients from Saudi Arabia, which could have been because the interviewer for the Saudi participants did not encourage the patients to comment further on their perceptions and attitudes toward diabetes insulin therapy. Nonetheless, studies with insulin-naïve patients including patients from Saudi Arabia with T2DM have reported that approximately 30% of the patients were unwilling to initiate insulin therapy if recommended because of various factors including perceptions of failure and self-blame [61-63]. Therefore, it is important that HCPs listen to and address patients’ concerns about insulin and provide accurate and adequate education on the progressive nature of T2DM and the ultimate need for insulin therapy at some point early on.

Although it has been established that illness perception interventions, often delivered over multiple sessions, can change perceptions and improve outcomes in T2DM [13,14], it is encouraging to find that a brief visual intervention (as short as 7 minutes) has the potential to change patients’ perceptions. This brief visual animation may improve current T2DM education and, if incorporated within larger illness perception interventions, could reduce intervention delivery time and therefore increase clinical utility.

This evidence is consistent with the findings from previous randomized controlled trials using different patient samples [37]. For example, both 3D models and an animation on osteoporosis increased perceived consequences, personal and treatment control, coherence perceptions and treatment motivation, and medication necessity beliefs, and decreased timeline perceptions and medication concerns [38]. A similar study found not only positive changes in perceptions (eg, increased treatment control and timeline perceptions and decreased identity perceptions) but also improved exercise and faster return to normal activities in patients with acute coronary syndrome compared with the control group [33]. Other visualization trials have reported improved adherence to antiretroviral therapy using viral load in patients with HIV [41,42] and increased postoperative mobility in oncology patients [43].

Family members’ perceptions have been shown to mediate relationships between patients’ perceptions and outcomes [30,31]. A recent review of CSM interventions in T2DM found that the inclusion of family members was important for improving patients’ glycemic control [32]. This study suggests that diabetes visualizations are also suitable for family members.

### Strengths and Limitations

A key strength of this study was the inclusion of 2 culturally specific versions of the visual animation and the cross-cultural patient samples. A second strength is the mixed methods design, which allowed us to gather both quantitative and qualitative data regarding the utility and acceptability of the visual animation. However, this study has a few limitations. First, patients and family members were not involved in co-designing the initial storyboards, scripts, or the visual animation assessed in this study; therefore, valuable input may have been missed. Second, because of convenience sampling, pre-post pilot design, and lack of a control group, our findings on changes in perceptions and self-efficacy must be interpreted with caution, and the study was not sufficiently powered to analyze scores by ethnicity (Māori, Pacific groups). Third, all patients perceived diabetes medications as necessary, and therefore it would be interesting to find out what a less receptive audience thinks about the visual animation and how it may influence their illness and treatment perceptions. Finally, data collection for this study was handled differently for the New Zealand and Saudi samples. Although extensive efforts were made to conduct the study completely on web, face-to-face recruitment from diabetes clinics was more successful in both countries. The New Zealand sample was comfortable using technology and therefore interviews were conducted over Zoom. Patients in the Saudi sample opted to perform the study in person at the clinic, either while waiting to be seen by the clinician or immediately afterward. In doing so, the opportunity for family members to participate was severely limited. Nonetheless, this study did help demonstrate the cross-cultural applicability of the visual animation. This also informs the practical aspects of recruitment for planning a future trial.

### Implications for Our Brief Visual Animation and Future Research

The brief visual animation will be adapted in light of the participants’ suggestions and feedback. This will include adding greetings in Te Reo Māori (for the New Zealand version), using a female Māori narrator voice, simplifying the language to overcome health literacy barriers, personalizing the animated characters to match the viewer’s gender and ethnicity, and adding subtitles to accommodate patients with hearing difficulties. The next step is to conduct a cross-cultural randomized controlled trial to investigate the effects on illness perceptions, adherence to medication, diet and exercise behaviors, glycemic control, and unplanned hospital admissions. The effect sizes found from this preliminary analysis will inform the sample size; therefore, the study will be sufficiently powered to conduct subgroup analyses by ethnicity. This future trial will ensure the recruitment of Saudi family members by offering incentives and allowing web-based participation for convenience. The inclusion of family members in the intervention is likely to have a larger impact on families and community health and improve the quality of life. This future trial could use the visual animation intervention as a stand-alone or as a component of a larger illness perception intervention. This future research would also look at how such an intervention could reduce or eliminate ethnic disparities and contribute to health equity.

## Appendix

Multimedia Appendix 1 Visual animation of type 2 diabetes mellitus.

Multimedia Appendix 2 Interview schedule and open-ended questions.

Multimedia Appendix 3 Patients’ and family members’ themes.

Multimedia Appendix 4 Health care professional’s themes.

## Abbreviations

B-IPQ: Brief Illness Perception Questionnaire
CONSORT: Consolidated Standards of Reporting Trials
CSM: common sense model
HCP: health care professional
T2DM: type 2 diabetes mellitus
