# Supplementary material for: Utility and Acceptability of a Brief Type 2 Diabetes Visual Animation: Mixed Methods Feasibility Study
Source: JMIR Form Res. 2022 Aug 9;6(8):e35079. doi: 10.2196/35079 (PMC9399876; doi:10.2196/35079)
Supplement: Multimedia Appendix 1 [file formative_v6i8e35079_app1.docx]

**Multimedia Appendix 1**

**Figure S1**. Patient character in the English version of the visual animation


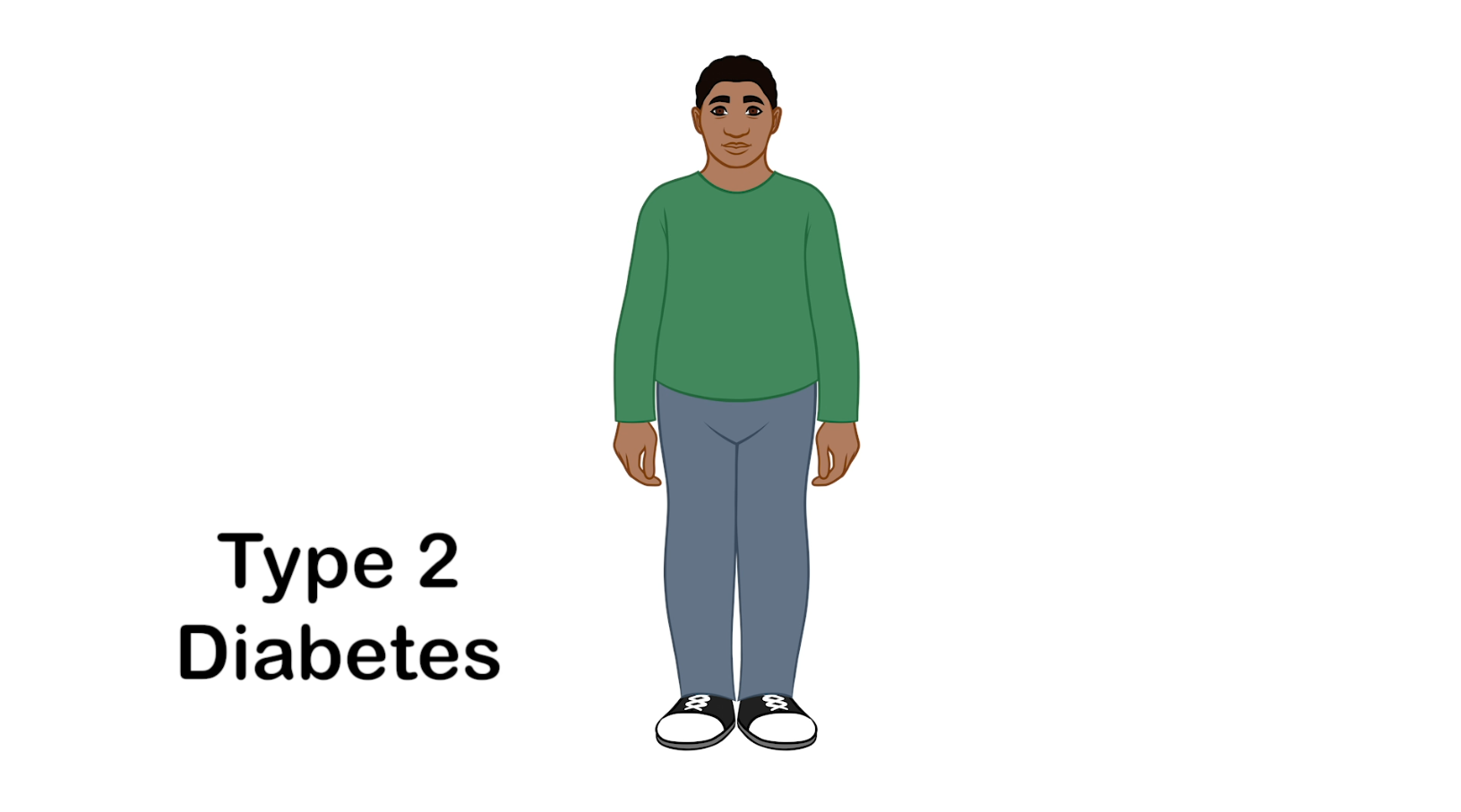


**Figure S2.** Patient character in the Arabic version of the visual animation


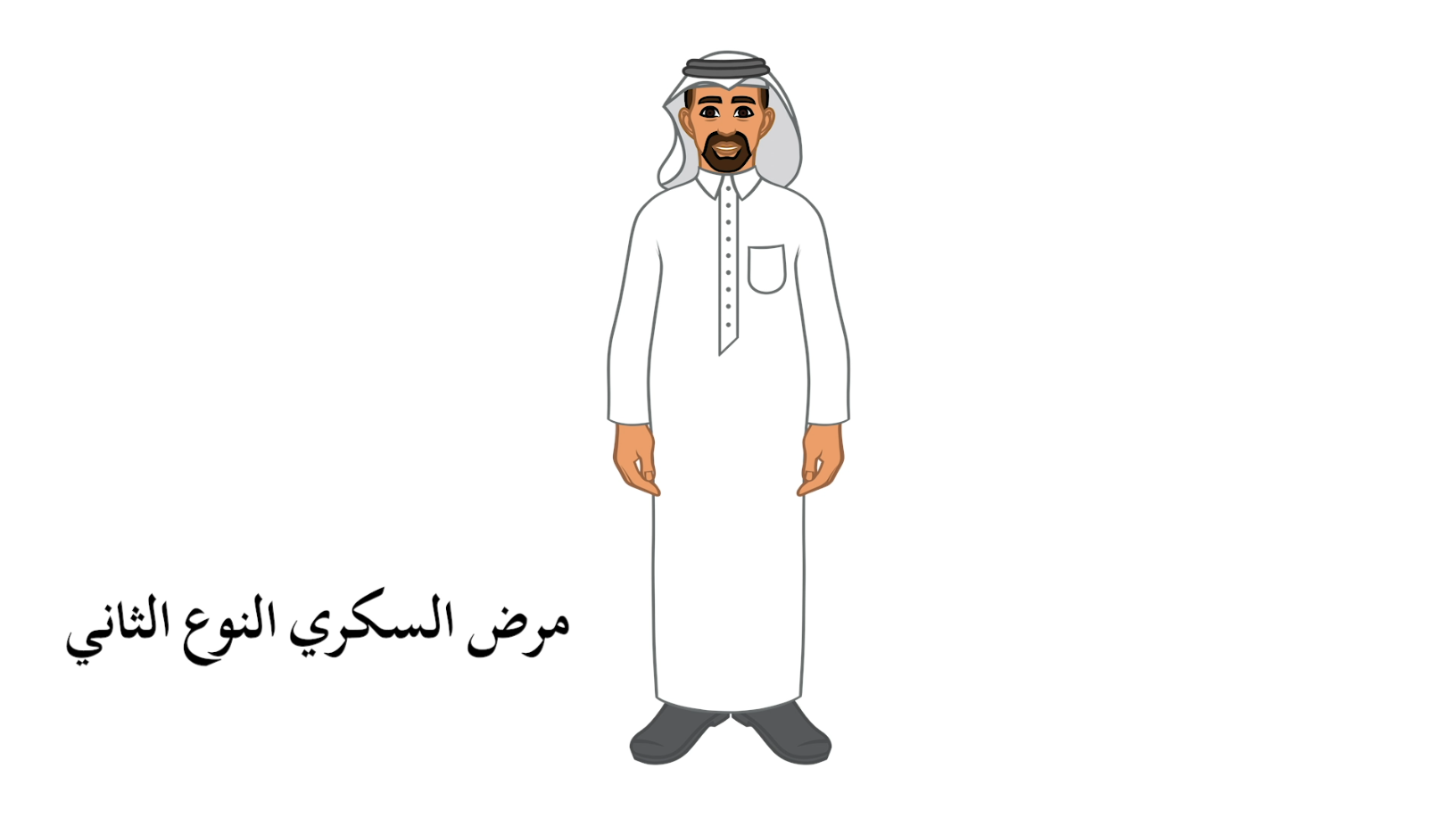


**Figure S3**. Glucose enters the bloodstream


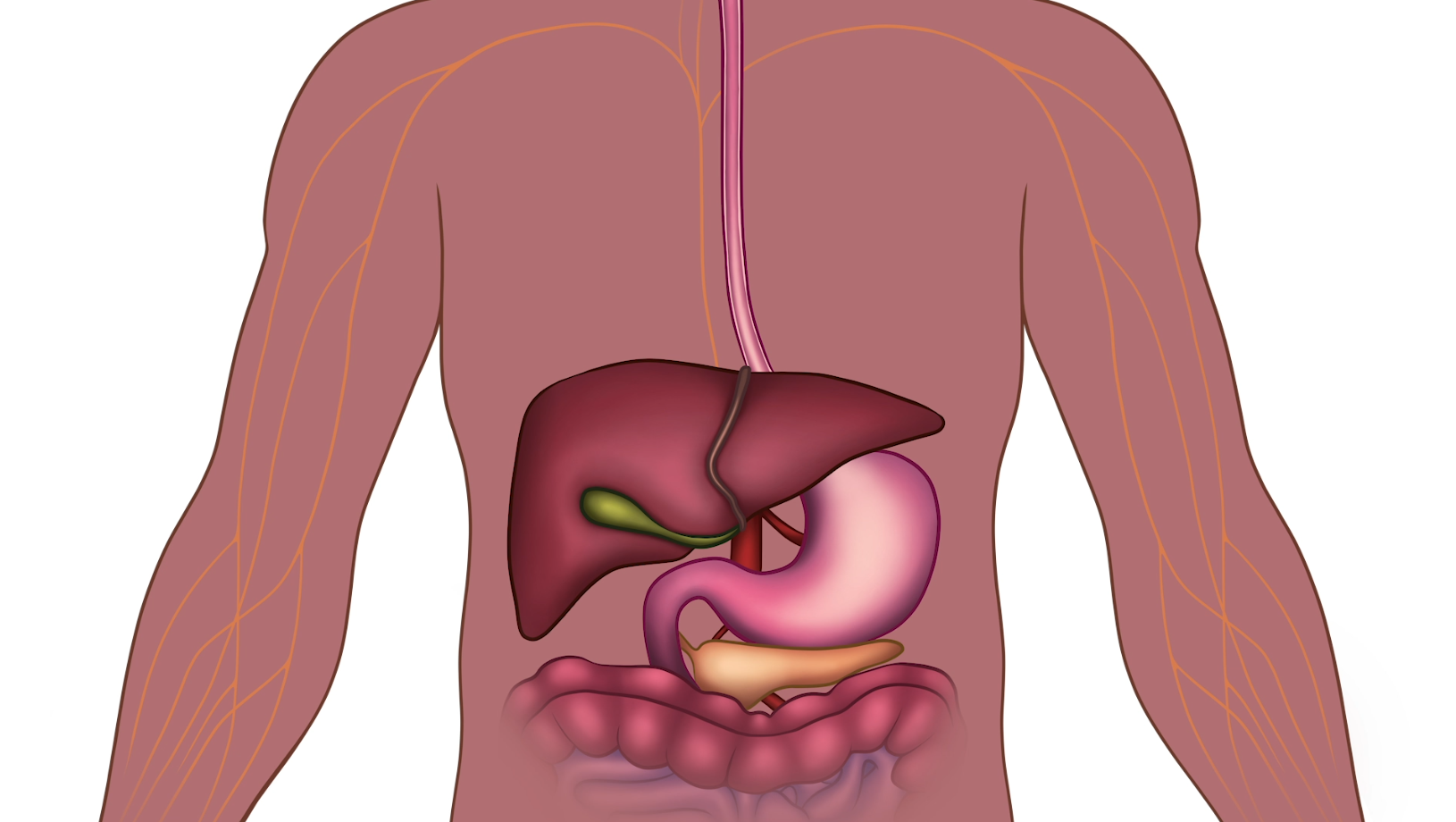


*Note*: This figure shows the inside of the human body. It explains how food is digested and then some of it is broken into glucose, which is a type of sugar that provides the body with energy. Glucose then goes into the blood and circulates through the bloodstream to reach body cells.

**Figure S4.** Glucose enters body cells to be used for energy


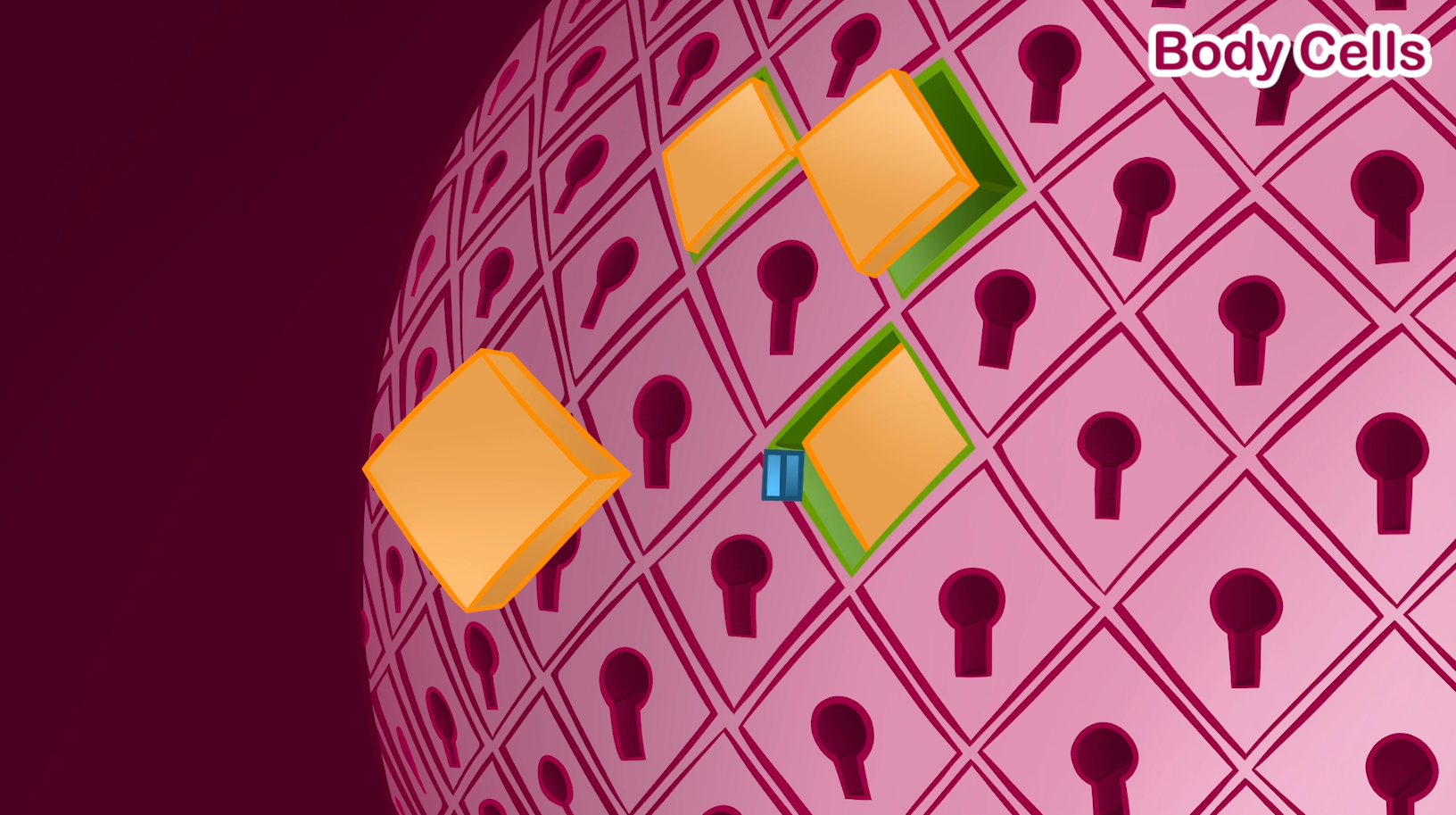


*Note*: This figure explains the importance of insulin (*blue rectangles*) for controlling the blood glucose (*yellow squares*) level. Insulin allows glucose to move from the blood to other cells of the body. Insulin acts like a key unlocking the door to let glucose into the cell.

**Figure S5.** Glucose builds up in the bloodstream with T2DM


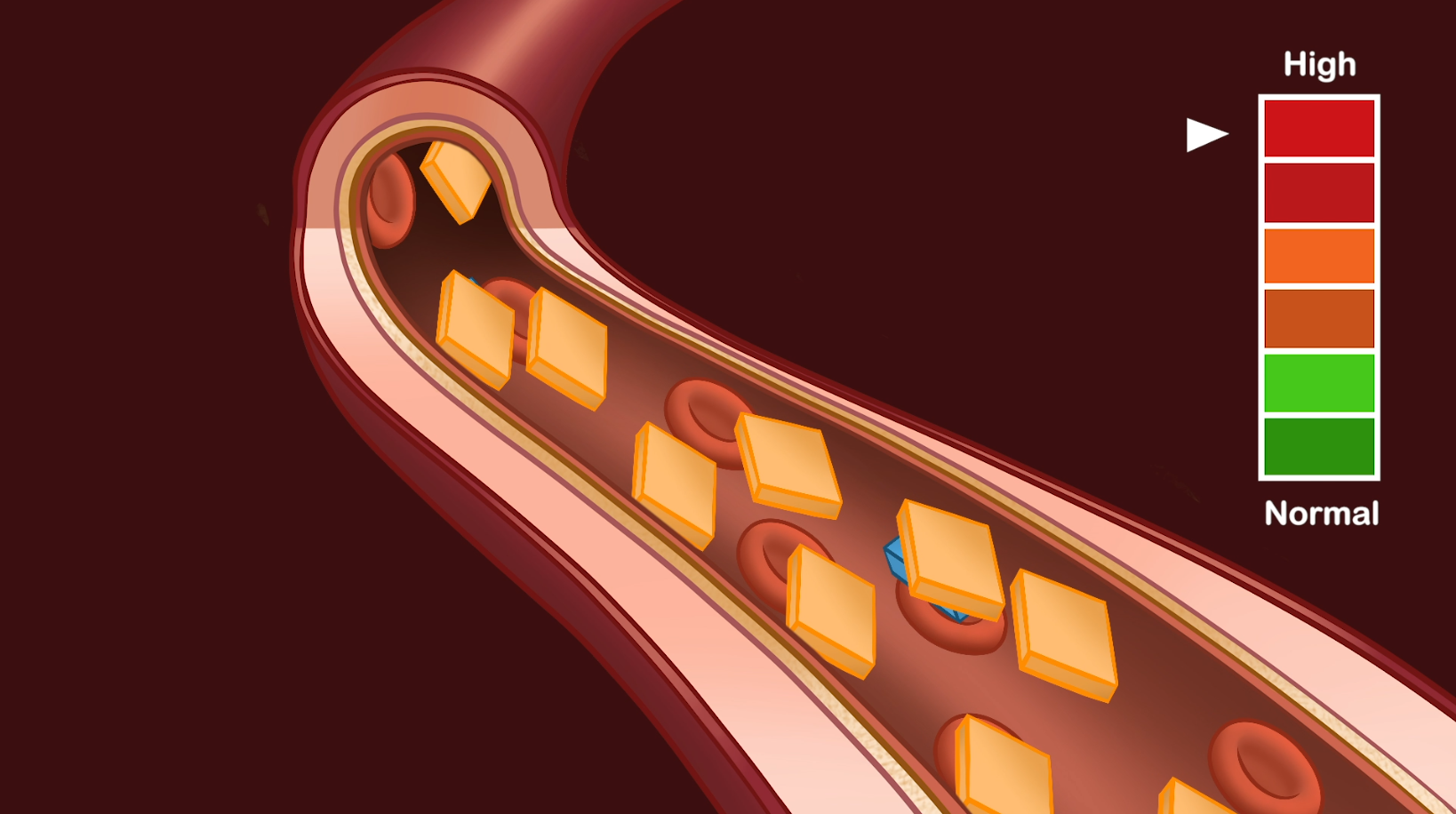


*Note*: This figure explains what happens with T2DM. It develops when the body is making inadequate amounts of insulin. T2DM can also develop when the body is unable to use the insulin it produces properly. The glucose cannot get from the bloodstream into the cells because there is not enough insulin to undo the lock to the cell doors. Blood glucose level remains high in the bloodstream (*represented by the gauge*).

**Figure S6.** Frequent infections and slow healing are among the symptoms of T2DM


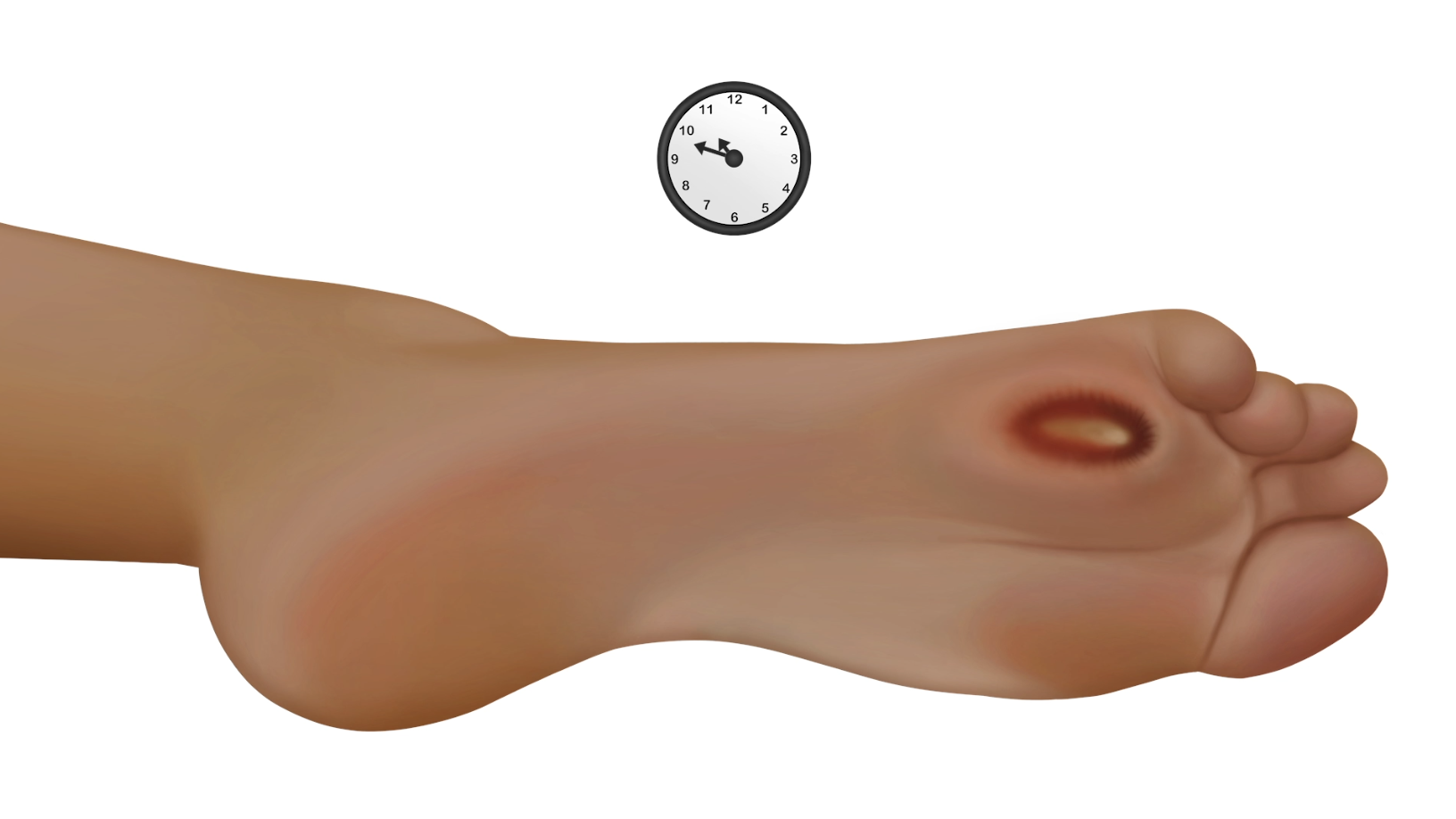


*Note*: This figure shows a foot infection/sore taking a longer time to heal (*indicated by the clock*).

**Figure S7.** Chronic complications of T2DM


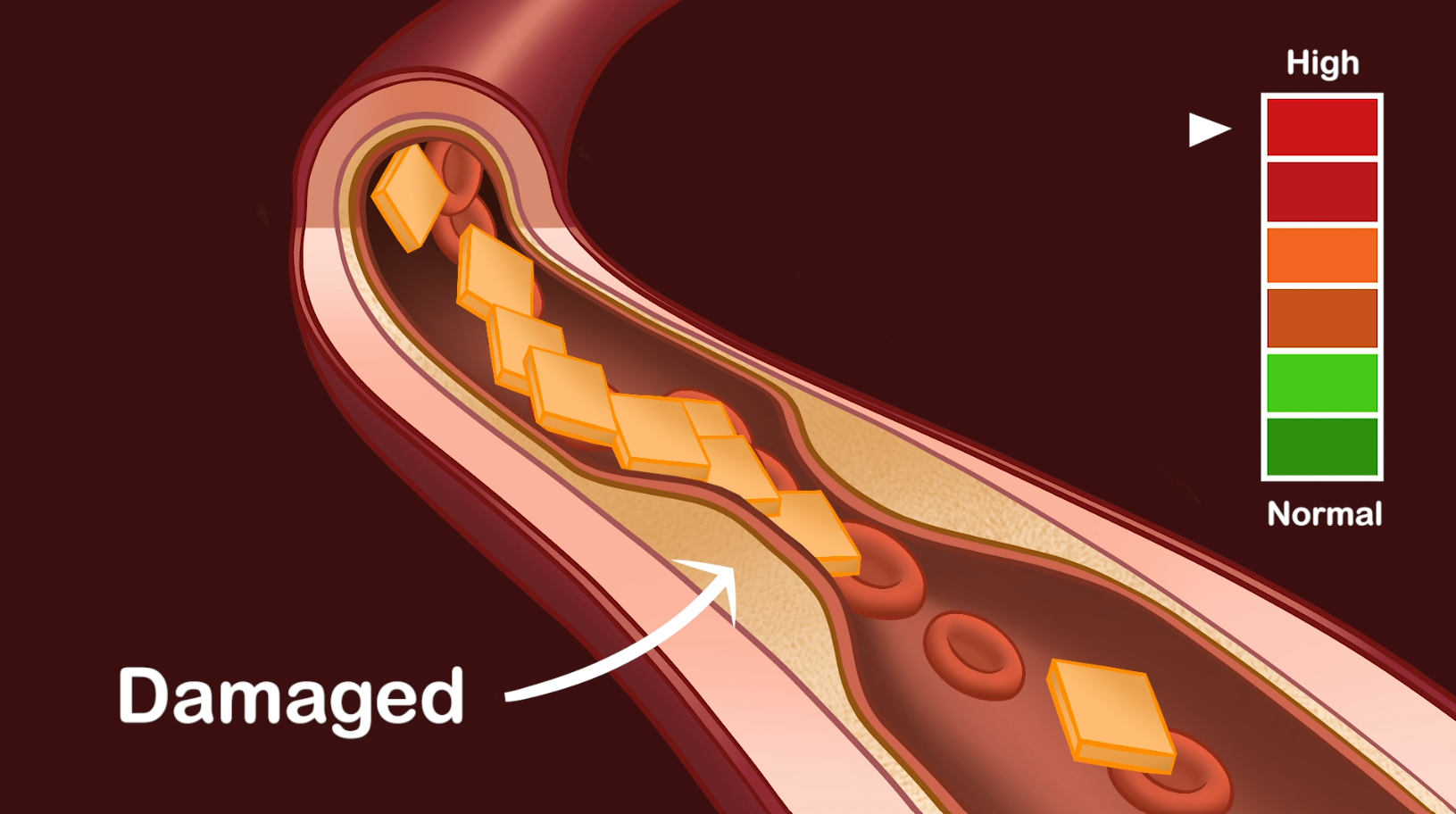


*Note*: This figure shows atherosclerosis (*narrowing of the arterial walls due to inflammation and plaque build-up*), which is a key pathological mechanism for macrovascular complications such as coronary heart disease and stroke.

**Figure S8.** Treatment for T2DM


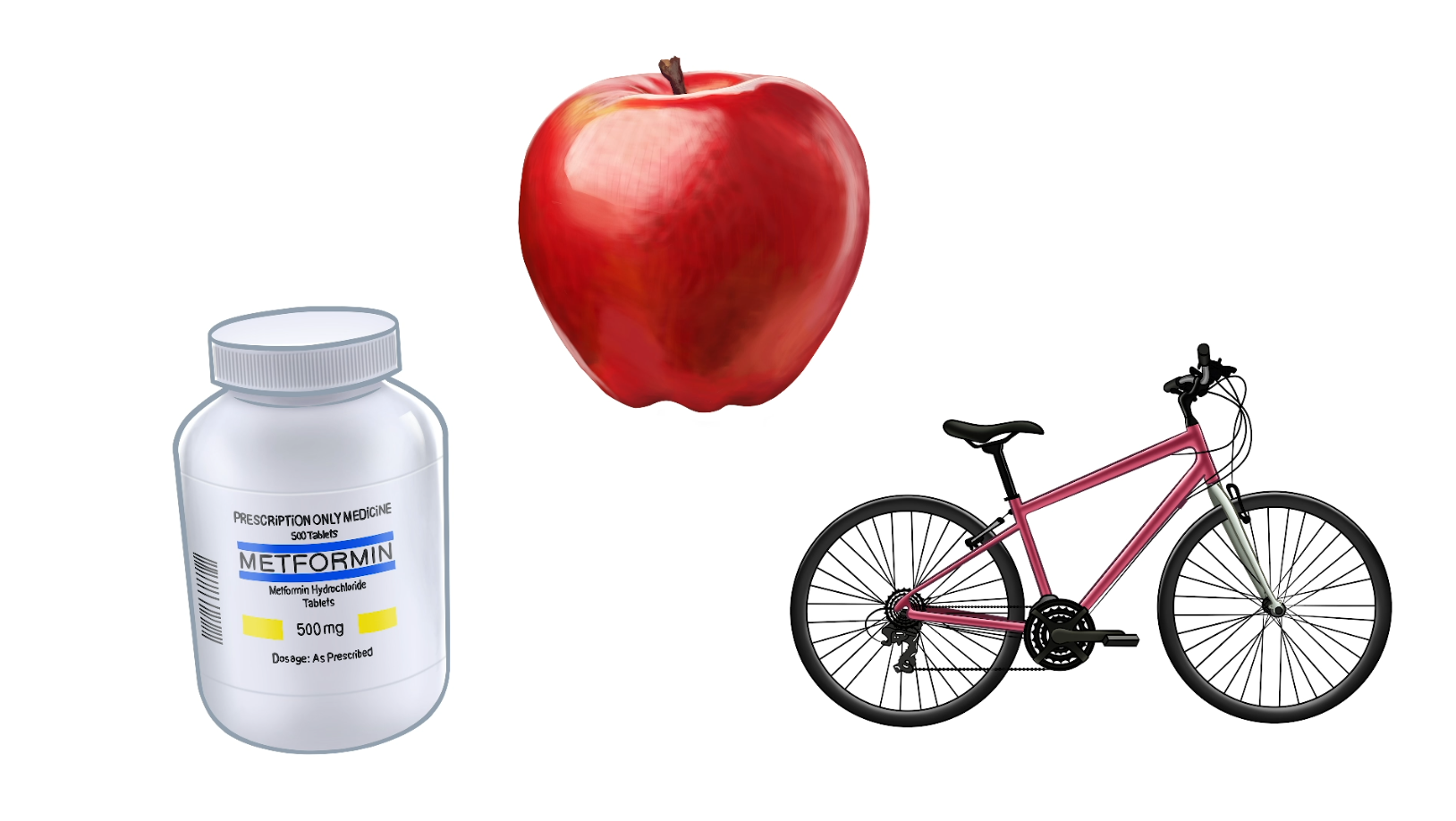


*Note*: This figure shows Metformin (*most commonly used medication for T2DM*), apple (*representing healthy diet*), and bicycle (*representing physical activity*).

**Figure S9.** Metformin helps regulate the amount of glucose in the bloodstream


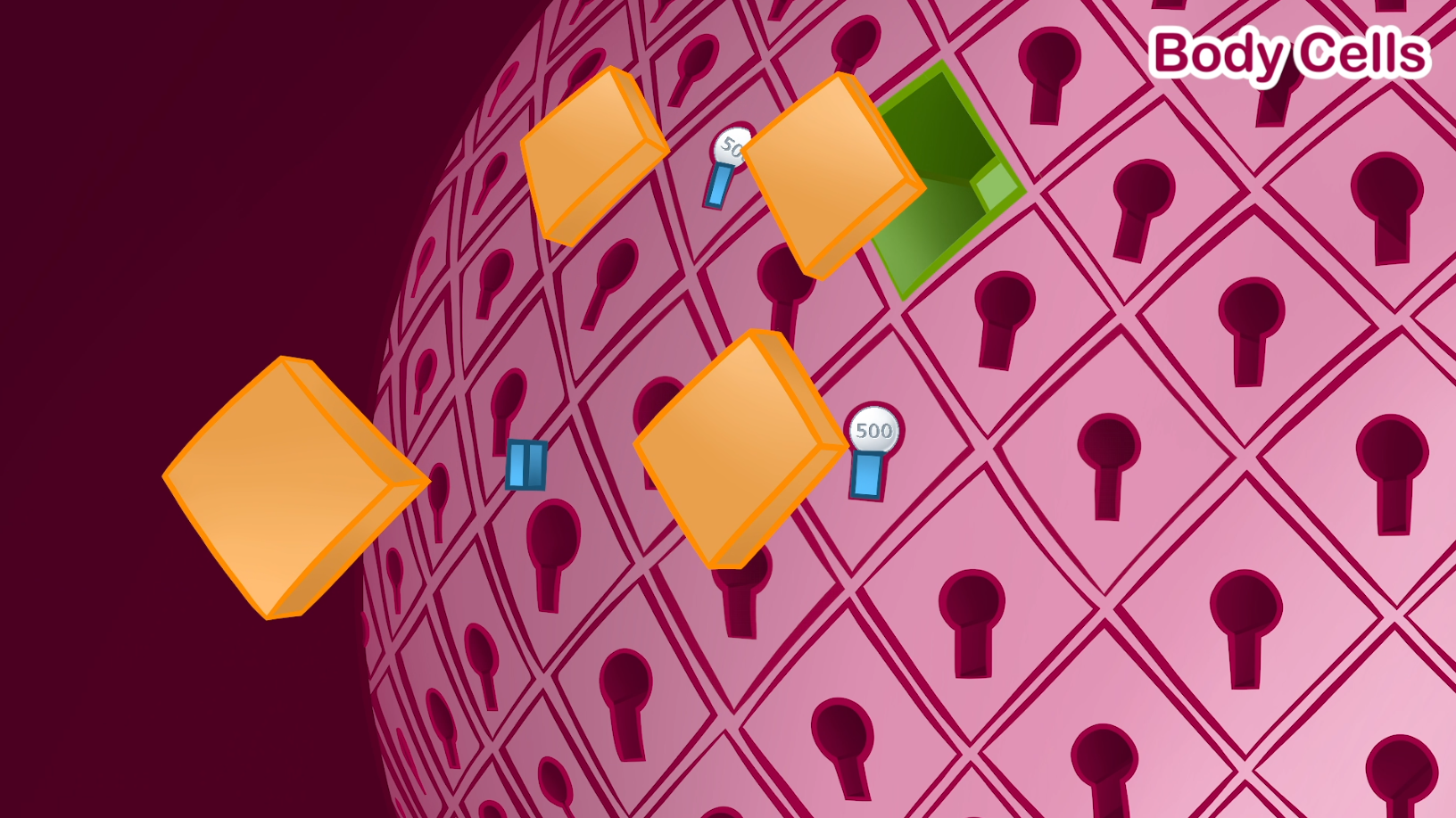


*Note*: This figure shows Metformin (*white 500 mg pill*) helping insulin (*blue rectangles*) regulate blood glucose level by decreasing insulin resistance and increasing insulin sensitivity and therefore allowing glucose (*yellow squares*) to enter body cells. Metformin also helps regulate blood glucose level by reducing the amount of glucose released by the liver.

**Figure S10.** Patient and family members in the English version of the visual animation


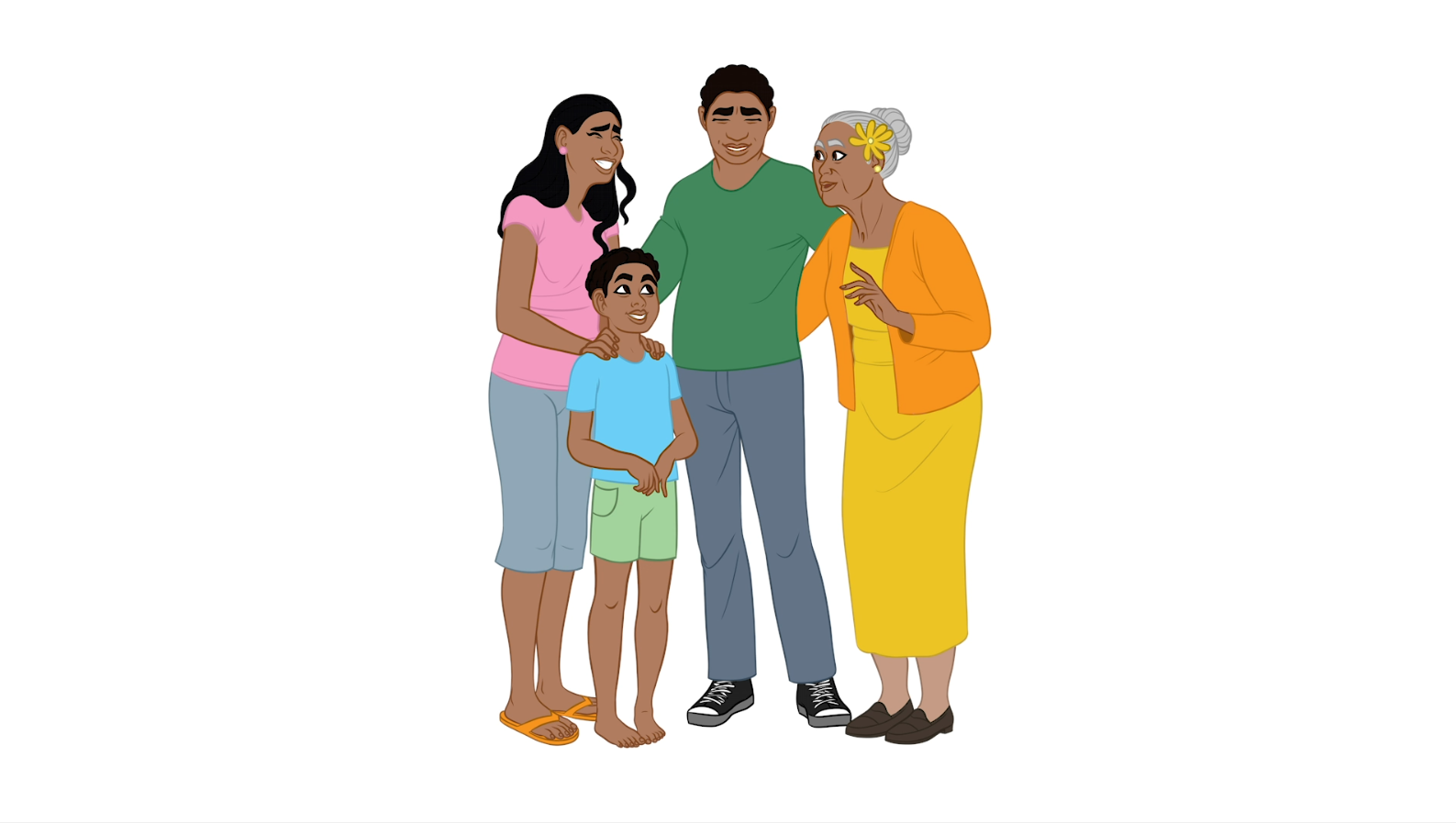


*Note*: This figure shows the patient with their family conversing and laughing. Family support is important as most of the diabetes self-care behaviours (e.g., healthy eating, exercising etc.) occur within the patient’s social context.

**Figure S11.** Patient and family members in the Arabic version of the visual animation


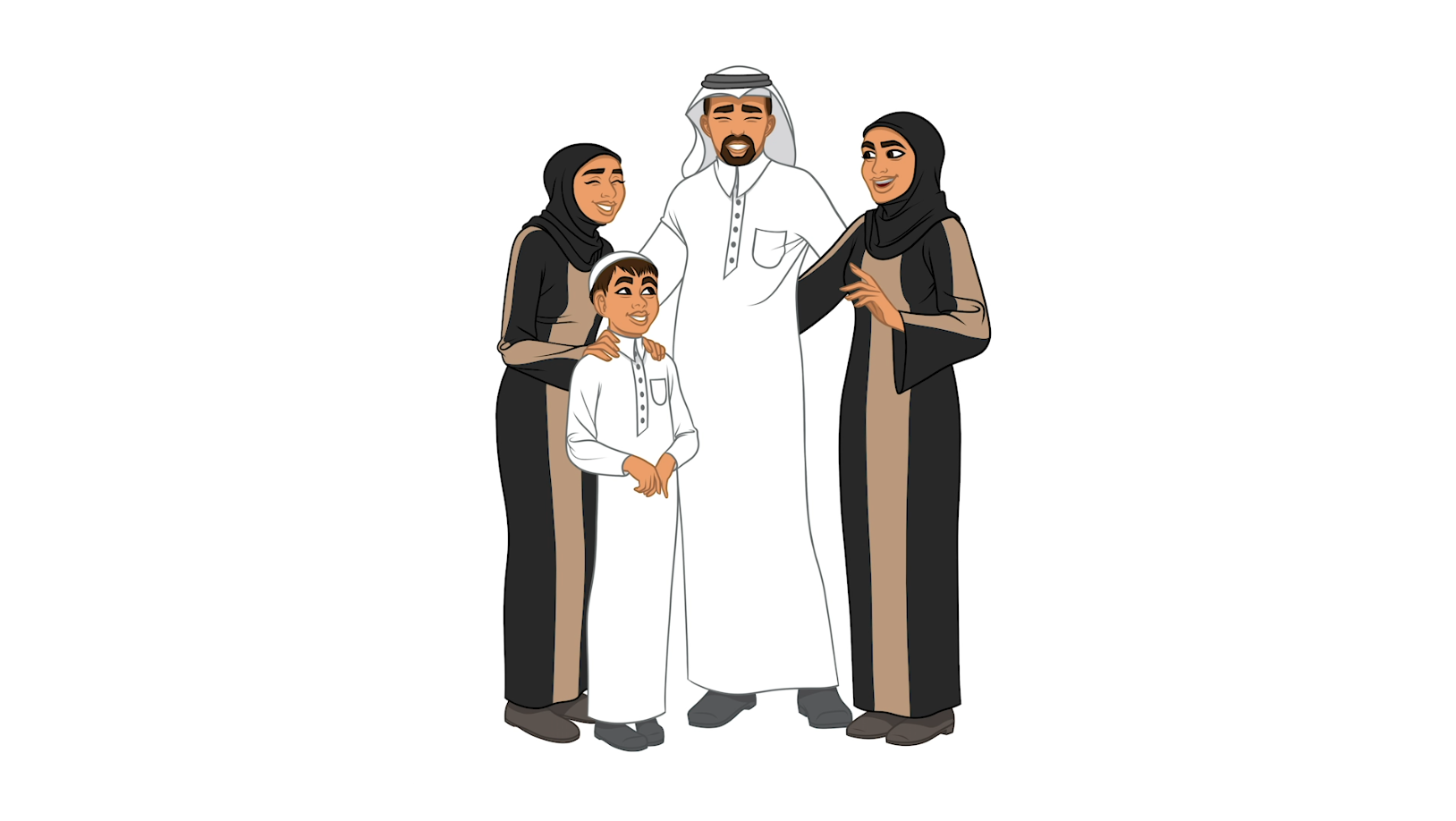


*Note*: This figure shows the patient with their family conversing and laughing. Family support is important as most of the diabetes self-care behaviours (e.g., healthy eating, exercising etc.) occur within the patient’s social context.
