# Supplementary material for: Utility and Acceptability of a Brief Type 2 Diabetes Visual Animation: Mixed Methods Feasibility Study
Source: JMIR Form Res. 2022 Aug 9;6(8):e35079. doi: 10.2196/35079 (PMC9399876; doi:10.2196/35079)
Supplement: Multimedia Appendix 2 [file formative_v6i8e35079_app2.docx]

**Multimedia Appendix 2**

1. **General structure and questions covered in the semi-structured interview with patients and family members**
2. Please tell me what you thought about this video?
3. Is there anything that the video said that did not make sense to you? Please explain
4. What went through your mind while you were watching the video?
5. Overall, was the animation easy to follow and understand?
6. Did the video help you understand what type 2 diabetes is, and how it affects the body? Which aspects of the video were the best?
7. Were there any aspects of diabetes that were not very well explained? What would you like explained better?
8. Did the video explain why it’s important to take medication and keep a healthy lifestyle? How could this be done better?
9. Does the video make you feel that diabetes is more manageable? Why/why not?
10. Do you have any concerns about your diabetes, your medication or lifestyle that you would like a future version of this video to include?
11. Was the content and language used easy to follow and understand?

No  Yes (please tell me more)

1. What did you think about the length and speed of the animation? (e.g. too long or too short/ too fast or too slow) Please tell me more
2. What did you think about the character? Can you relate or connect to them at all? Why or why not?
3. Do you think it would be better if the animated character matched your sex? (e.g. a female character for a female participant)

No  Yes (please tell me more)

1. What would you change in the video to make it better? Please tell me more
2. Is there anything else you would like to comment on about the video? Please tell me more
3. What other information have you seen about type 2 diabetes (e.g., in hospital pamphlets or online)?
4. How does this video compare to what you have seen before?
5. **Open-ended questions for healthcare professionals**

We would like to ask you a few questions about your views of the brief diabetes animation you just watched. This will help us create the alpha version of the animation. There are no right or wrong answers­­ to any of these questions; we are only interested in your opinions.

1. What is your role?

Endocrinologist

Nurse

Dietitian

Diabetes educator

Health Psychologist

Podiatrist

Other, please specify …………………………...

1. Was the content and language used easy to follow and understand?  Yes  No

Comment:-------------------------------------------------------------------------------------------

1. What did you think about this animation?

Comment:-------------------------------------------------------------------------------------------

1. Do you think this animation would help patients and their whānau to better understand diabetes?

Comment:-------------------------------------------------------------------------------------------

1. Is animation a good way to deliver this education to patients and their whānau?

Comment:-------------------------------------------------------------------------------------------

1. Do you think it’s suitable for Maori and Pacific patients with type 2 diabetes?

Comment:-------------------------------------------------------------------------------------------

1. What would you change in the animation to make it better?

Comment:-------------------------------------------------------------------------------------------

1. Is there anything else you would like to comment on about the animation?

Comment:------------------------------------------------------------------------------------------
