# Supplementary material for: Utility and Acceptability of a Brief Type 2 Diabetes Visual Animation: Mixed Methods Feasibility Study
Source: JMIR Form Res. 2022 Aug 9;6(8):e35079. doi: 10.2196/35079 (PMC9399876; doi:10.2196/35079)
Supplement: Multimedia Appendix 3 [file formative_v6i8e35079_app3.docx]

**Multimedia Appendix 3**

*Themes and sub-themes identified from interviews with patients and family members with example quotations*

| Theme and subtheme | | Example quotes^a^ |
| --- | --- | --- |
| **Animation-related factors** | |  |
|  | Succinct and simple | *This one [animation] was simple, short and catches the attention.* [SA^b^ male patient 7, 44 years]  *It was very succinct, and it explained all the different parts that you need to think about diabetes fairly well, especially for someone who doesn't want to get bogged down in the details.* [NZ^c^ female family member 3, 48 years] |
|  | Understandable | *I thought it was probably the most straightforward bloody thing I've seen. Some of the other stuff are very confusing.* [NZ female patient 6, 40 years]  *The part of insulin’s role in controlling blood glucose was well animated.* [NZ female family member 12, 42 years] |
|  | Informative | *That bit about the lock and key, that was really good. Because I didn't understand it before. I’ve had it for over 10 years, and I could never work out how it worked and with the key that sort made more sense.* [NZ male patient 14, 51 years]  *The video was clear in explaining the causes of diabetes and how treatment can help, which I appreciate.* [SA male patient 16, 39 years]  *I think that it was very informative because you know like the specifics of what actually goes on in the body you know, 'cause you kinda know but you don't.* [NZ female family member 3, 48 years] |
|  | Character factors | *I identified with him a lot; it wasn't typical stuff in the brochures… I don't know if I internalized it so much as thought oh that's what's going on in me… He had facial expressions that made him real… not just a flat character.* [NZ female patient 6, 40 years]  *I liked the characters. I felt connected to them, because they were characters from my culture.* [SA female patient 6, 59 years]  *As soon as the male figure came on, your mind goes well this doesn't associate to me does it? Yeah, this is a male, we are different.* [NZ male patient 14, 51 years]  *Diabetes involves everybody, and the character is just an image that you are using to get the message across.* [NZ male patient 18, 62 years] |
| **Impacts of the animation** | |  |
|  | Patient reflections | *The video made me think about my diabetes and how I can do better to control it.* [NZ female patient 9, 54 years]  *That's what changed my whole mindset while I was watching your video. So, if I go back to the doctor in two months from now, and they say you're still high, I'm gonna feel much more encouraged about insulin now.* [NZ male patient 10, 36 years]  *Before seeing that video, in my mind, diabetes was all about ‘ohh don't get an insulin shot and gotta have one of those every day’, but the video was really good at explaining you know, it put a few things in my mind at rest.* [NZ male patient 18, 62 years]  *That part of the animation reinforced what I really should be doing to get my blood sugar down and basically just having a healthy life and living with diabetes type 2.* [NZ female patient 17, 54 years]  *I thought about the importance of exercise and eating healthy and that I have to engage in these two things more than what I am doing now.* [SA male patient 3, 38 years]  *I felt guilt when you had the bicycle there, was like it's been cold and raining and I haven't been out as much as I usually do.* [NZ female patient 6, 40 years] |
|  | Family members’ reflections | *It was good reminder to me on what I can be doing to support them… I can definitely do things day-to-day, which is good and that's what I was reflecting as the video was playing.* [NZ female family member 11, 30 years]  *When it said you know diet or exercise, it made me think about whether or not they're doing it.* [NZ female family member 3, 48 years] |
| **Animation as an effective format** | |  |
|  | Visual information | *You know the written words; you know what the doctor is saying, and you'll see a lot of people that from the Pacific and Māori communities they will just sit there and nod yes at the doctor because they don't understand. But being a bit shy, not wanting to express themselves, they'll just sit there and nod yeah because they don't understand what's being told to them.* [NZ female patient 17, 54 years]  *Visual is always better than written one, because in the visual you actually see what's happening.* [NZ female family member 16, 57 years)  *I learnt a lot, things that have been explained to me before by the doctor and the education nurses, but never like this visually.* [SA male patient 4, 56 years]  *The video is more engaging for sure, no doubt about it. Seeing things visually makes me understand more.* [SA male patient 12, 55 years] |
|  | New information | *I also thought I should have known all this info you know; I’ve been living with diabetes for so long.* [NZ male patient 8, 71 years]  *It discussed a lot of things, some of which I had already known, and other points I didn’t know about before* [SA female patient 8, 41 years] |
| **Diabetes management-related factors** | |  |
|  | Barriers to adherence | *I forget my medication sometimes, you know, when you get old, you forget things.* [NZ female patient 13, 59 years]  *I work 80 hours, so it is hard to eat and take the meds at the same time. Sometimes I get home late, and I usually remember to take my meds, it’s just sometimes I forget.* [NZ female patient 2, 61 years]  *I used to skip my medications sometimes, because I feel okay.* [NZ female, patient 9, 54 years]  *Sometimes you just indulge in things that you wanted to eat because of what's happening around you.* [NZ female patient 22, 48]  *I can start going for a walk but if it rains, ‘ohh it's raining’ so I won't bother.* [NZ female patient 5, 62 years] |
|  | Concerns and frustrations | *I am a bit concerned about the side effects of the medication, especially the insulin.* [SA male patient 7, 44 years]  *In my mind if I go on insulin, I have failed.* [NZ male patient 15, 56 years)  *When he saw the nurse, she laughed at [him] and she said ‘ohh doctor Google, oh doctor Google.* [NZ female family member 11, 30 years]  *Although strokes and heart attacks can happen later on in life, diabetes can perhaps bring them on earlier if you don't control it.* [NZ male patient 15, 56 years] |
|  | Cultural influences | *In the context of Pacific and Māori. So, if you're out for the day getting something to eat, then you go to a restaurant like Taco Bell for an example you know. You're considered to be rich or someone who's really impressive if you get the large combo and the large combo always comes with a large coke, so you've got the status problem with people wanting to get a large because they're wanting to be seen as like impressive.* [NZ male patient 10, 36 years]  *I should take care of what I eat more I admit, but you know our culture, sometimes it is hard or even impossible to say no.* [SA female patient 15, 68 years] |
|  | Importance of support | *Best for me was the family help, I think. Get your family help to stick to the exercise and diet. My kids helped me a lot, and make sure I'm eating properly, they are always on me.* [NZ female patient 2, 61 years]  *If they're [patients with T2D] kind of stuck in their ways… I think it's going to be harder for you to get them to do something about it if they don't want to do something about it.* [NZ female family member 1, 32 years]  *My husband doesn't worry about diabetes, so it's quite hard in the eating area in my household...the people around me can eat whatever they like. That can be quite challenging, it's hard for me.* [NZ female patient 5, 62 years]  *In regard to the family, it is important that the whole family is educated about my illness. This makes dealing with diabetes much easier.* [NZ male patient 8, 71 years] |

^a^Quotes are reported verbatim.

^b^SA: Saudi Arabia.

^c^NZ: New Zealand.
