# Supplementary material for: Utility and Acceptability of a Brief Type 2 Diabetes Visual Animation: Mixed Methods Feasibility Study
Source: JMIR Form Res. 2022 Aug 9;6(8):e35079. doi: 10.2196/35079 (PMC9399876; doi:10.2196/35079)
Supplement: Multimedia Appendix 4 [file formative_v6i8e35079_app4.docx]

**Multimedia Appendix 4**

*Themes and sub-themes identified from health care professionals’ responses with example quotations*

| Theme and subtheme | | Example quotes^a^ |
| --- | --- | --- |
| Animation-related factors | |  |
|  | Brief and succinct | *Very clear, concise, effective.* [Staff 9, medical student] |
|  | Easy to understand | *It was good not to have a lot of medical terms.* [Staff 10, nurse]  *Helps them [patients] visualize concepts and retained in visual and audial memory… I especially liked the "keyholes in the cell wall" for illustrating the action of insulin and Metformin.* [Staff 11, medical student] |
|  | Informative | *Gives good general view about diabetes for people who do not know anything about what diabetes is.* [Staff 2, nurse]  *Simple, initial video suitable for those with recent diagnosis and poor/early education needs.* [Staff 5, endocrinologist] |
|  | Culturally appropriate | *Main character looks physically more representative of this population. Hopefully, more relatable.* [Staff 11, medical student] |

^a^Quotes are reported verbatim.
